# Developing Modern Pain Therapies

**DOI:** 10.3389/fnins.2019.01370

**Published:** 2019-12-20

**Authors:** John Manion, Matthew A. Waller, Teleri Clark, Joshua N. Massingham, G. Gregory Neely

**Affiliations:** ^1^The Dr. John and Anne Chong Lab for Functional Genomics, Charles Perkins Centre and School of Life and Environmental Sciences, The University of Sydney, Sydney, NSW, Australia; ^2^Genome Editing Initiative, The University of Sydney, Sydney, NSW, Australia

**Keywords:** pain, neuropathy, inflammation, dysfunctional, analgesia, cell therapy, gene therapy, opioids

## Abstract

Chronic pain afflicts as much as 50% of the population at any given time but our methods to address pain remain limited, ineffective and addictive. In order to develop new therapies an understanding of the mechanisms of painful sensitization is essential. We discuss here recent progress in the understanding of mechanisms underlying pain, and how these mechanisms are being targeted to produce modern, specific therapies for pain. Finally, we make recommendations for the next generation of targeted, effective, and safe pain therapies.

## Introduction

Pain represents a major challenge to modern medicine. Chronic pain is the most common neurological disorder and migraine, a subtype of pain, is the most common reason for presentation to a neurologist. Chronic pain is exceptionally disabling, and assessments of the global burden of disease consistently rank headache and low back pain as the diseases causing the greatest global disability burden (Menken et al., 2000; GBD 2016 Neurology Collaborators et al., 2019). Chronic pain affects a large number of individuals with estimates of prevalence of chronic pain (all forms) up-to 50% with severely disabling pain affecting up to 14% of the population (Fayaz et al., 2016) although estimates of prevalence vary considerably (Dahlhamer et al., 2018). Pain medications remain amongst the most commonly prescribed treatments (AHRQ, 2019) and pain ranks as the primary reason patients suffering any disease present to their doctors (Schappert and Burt, 2001). Pain is defined by the International Association For The Study of Pain as “an unpleasant sensory and emotional experience associated with actual or potential tissue damage, or described in terms of such damage” (IASP, 2012) although it will likely be updated to reflect that resemblance to this state should be sufficient and that an inability to communicate is not reflective of a lack of pain (IASP, 2019). Pain provides a reinforcing stimulus to prevent damage and an affective emotional component to encourage avoidance of damaging stimuli in the future. Whilst our bodies need a system to detect damage in order to protect ourselves from such damage, the system is highly susceptible to pathological sensitization causing substantial disability. Given the current crisis in pain therapy, we examine the hallmarks of pathological transitions to chronic pain with particular focus on peripheral and spinal mechanisms and finally we examine recent efforts to generate long-lasting, local therapies for pain by targeting these mechanisms.

## Pain Stratification

Pain is currently stratified into chronic, subacute, and acute pain. Chronic pain is defined as persistent or recurrent pain that lasts longer than the typical healing process often arbitrarily considered 3 months; acute pain being that lasting under 6 weeks and subacute pain lasting between 6 and 12 weeks (IASP, 2012). Since acute to chronic pain transitions occur early in pain pathogenesis in preclinical models, it is unclear if these distinctions are useful for patients (Bennett and Xie, 1988; Decosterd and Woolf, 2000; Price et al., 2018). Additionally, pain can be further stratified into three distinct groupings based on the assumed mechanism of action and these groupings can dictate treatment in some cases (Costigan et al., 2009). These are dysfunctional, inflammatory (caused by inflammatory conditions) and neuropathic pain (caused by nervous system damage). Dysfunctional pain is possibly the most poorly understood where there is no clear lesion to the central nervous system or inflammation that can adequately explain the pain. A breakdown of these conditions and current treatment strategies is provided in Table 1. Chronic pain conditions cause a variety of symptoms, particularly hyperalgesia, where painful stimuli are perceived as more painful than they actually are, and allodynia where normally non-noxious stimuli elicit a pain response. Given differential treatment efficacy of analgesia for pain subtypes, further stratification of pain disorders may be highly desirable (Themistocleous et al., 2018).

**TABLE 1:** Stratification of pain by cause.

Type of pain	Examples	Treatment strategy
Neuropathic	Trigeminal neuralgia, post-herpetic Neuralgia, trauma, Chemotherapy induced neuropathy, antibiotic induced neuropathy, diabetic neuropathy, hereditary neuropathies, sciatica, anti-retroviral induced neuropathy, multiple sclerosis, tumors (Freynhagen and Bennett, 2009).	Removal of inciting agents for chemotherapy and antibiotics (dose-limiting) tri-cyclic antidepressants such as nortriptyline, Serotonin-Noradrenaline Re-uptake inhibitors such as duloxetine Anticonvulsants including lamotrigine and carbamazepine. Surgical options for lesions. Opioids in some cases for intractable pain (Freynhagen and Bennett, 2009).
Inflammatory	Rheumatoid Arthritis (Lee, 2013), osteoarthritis, gout (Roddy et al., 2013), myositis, Sjogren’s syndrome (Holdgate and St Clair, 2016), systemic lupus erythematosus, tumors (Schmidt et al., 2010).	Anti-inflammatories, opioids, disease modifying antibodies such as Anti-TNF, surgical approaches to remove underlying sources of inflammation.
Dysfunctional	Bladder pain syndrome (Offiah et al., 2013), irritable bowel syndrome, paroxysmal extreme pain disorders, primary erythermalgia, temporomandibular disorder, and fibromyalgia (Crabtree and Ganty, 2016).	Few, shares features of neuropathic and non-neuropathic pain, exercise therapy, NSAIDs, anti-depressants including TCAs, monoamine oxidase inhibitors, SSRIs and SNRIs, opioids, and anti-convulsants are used with varying levels of efficacy.

### Inflammatory Pain

Inflammatory pain disorders include pain caused by inflammation generally elicited by some kind of noxious insult or degeneration for example the disorders of rheumatoid arthritis, osteoarthritis and after tissue trauma where inflammatory mediators and immune cells are recruited and secrete inflammatory mediators directly causing peripheral sensitization and later central sensitization. Inflammatory disorders are more commonly associated with hyperalgesia rather than allodynia. These conditions remain highly prevalent and are becoming more so with an aging population given that many are age-related conditions. Inflammatory pain is likely a protective phenomenon; when injured it is necessary to prevent use of the affected area to promote healing.

### Neuropathic Pain

Neuropathic pain disorders are among the most treatment resilient pain conditions. Unlike inflammatory pain, neuropathic pain appears entirely maladaptive. Neuropathic pain by definition involves lesion or inflammation of the nervous system and includes diseases such as trigeminal neuralgias, peripheral neuropathies, and post-herpetic neuralgia. The uniting factor is the etiology of lesion to peripheral or central nervous system (IASP, 2012), although in some cases of clinical neuropathic pain there is no obvious lesion, for example many cases of trigeminal neuralgias (Ko et al., 2015). Neuropathic pain is generally identified by its specific character, neuroanatomical distribution, plausibility, lesion confirmation and co-occurrence with disorders such as diabetes mellitus (Themistocleous et al., 2018). The character of neuropathic pain is described as sharp, shooting pain with similarity to electric shocks. The sensations of tingling, pins and needles, and alternating numbness are also very common. Neuropathic pain is particularly associated with touch allodynia where innocuous touch stimuli become excruciatingly painful and this can occur with very minor stimuli including the touch of clothing or the wind (Woolf and Mannion, 1999).

### Dysfunctional Pain

Dysfunctional pain disorders include bladder pain syndrome (previously interstitial cystitis) (Offiah et al., 2013), irritable bowel syndrome (IBS), and fibromyalgia. A tentative definition is: pain disorders associated with abnormal functioning of the somatosensory system (Costigan et al., 2009) These are pain conditions associated with a lack of detectable inflammation or tissue damage and an obvious mechanism (IASP, 2012). A unifying feature amongst these conditions is, with the notable exception of fibromyalgia, their location in viscera rather than affecting afferents of the skin. Given the focus of research on primary skin afferents, it is perhaps unsurprising we have a more limited understanding of these conditions. Dysfunctional pain has thus far been a defined grouping of potentially loosely associated disorders and it is unclear whether the lack of detectable inflammation, nervous system damage, or acute noxious stimuli may be more a consequence of insensitive tools rather than reflective of the nature of the condition. Indeed in the case of interstitial cystitis more recent evidence from biopsy gene expression analyses suggests patients suffer from an inflammatory condition in the bladder (Offiah et al., 2016). Dysfunctional pain disorders often entail a mix of pain behaviors including multimodal allodynia and hyperalgesia. Given our lack of understanding and previous debates as to “the existence” of central pain disorders (Wolfe et al., 2009); it is perhaps unsurprising that there are few effective treatments for these diseases.

## Peripheral Sensitization

**FIGURE 1:**
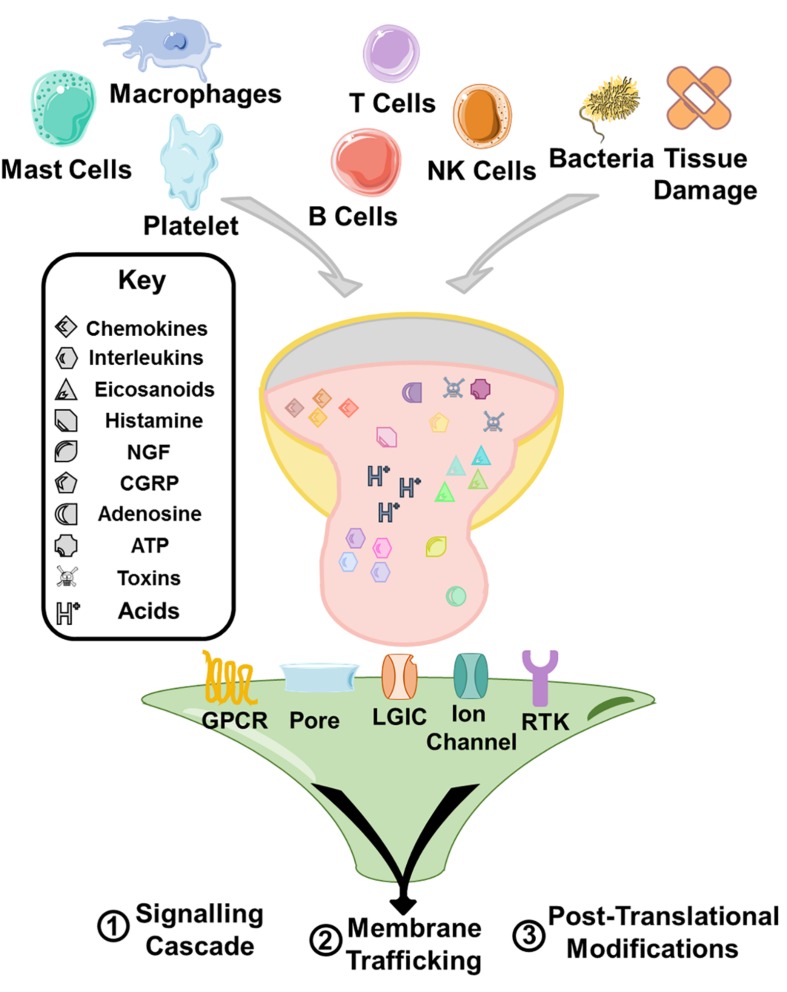
A range of immune cells, direct release from tissue, toxins, and bacteria contribute an inflammatory milieu that sensitizes immune cells. The principle mechanisms of sensitization are through modulation of signaling cascades triggering various cellular responses, and upregulation of trafficking of receptors to the membrane. Furthermore, receptor activation can also lead to post-translational modification of receptors and ion channels and the modulation of their activity causing sensitization. The receptor activation by the inflammatory soup is triggered principally through G protein coupled receptors (GPCRs), receptor tyrosine kinases (RTKs), and ligand gated ion channel type receptors (LGIC/iontropic receptors or the modulation of ion channels that underlie the intrinsic excitability of cells such as nav1.7.

Peripheral sensitization can be a feature of any type of pain. It is defined as any increase in the responsiveness of the nociceptive circuit at the level of the periphery and a lowering of the threshold at which pain is perceived. Primarily, there is the peripheral release of the ‘inflammatory soup’ (Figure 1) consisting of an assortment of different mediators. The principle sources of these molecules are the resident mast cells, and infiltrating neutrophils, macrophages, platelets, other immune cells and direct releases from tissue damage, however it is also increasingly recognized that non-host factors including bacterial toxins also play a direct role in this process (Basbaum et al., 2009). These mediators include histamine, adenosine triphosphate (ATP), nerve growth factor (NGF), substance P, bradykinin, calcitonin gene-related peptide (CGRP), various cytokines, prostaglandins, adenosine, and acids. During activation of nociceptors by the mediators, the properties of nociceptive ion channels (that sense or modulate pain transmission) such as TRPV1 (Caterina et al., 1997), TRPA1 (Bautista et al., 2006), Nav1.7 (Cox et al., 2006), Nav1.8 (Faber et al., 2012), and Nav1.9 (Dib-Hajj et al., 2015) are altered via phosphorylation or alternative mechanisms, leading to increased neuronal activity, reduced activation thresholds or increased currents (Pinho-Ribeiro et al., 2017).

### Sources of the Soup

#### Innate Immunity

The innate immune system has a major role in driving nociceptive hypersensitivity. Macrophages, a major component of innate immunity, plays dual roles during injury: they act to promote inflammation, and drive the immune response to non-host factors (in the M1 macrophage state) triggered by interferons, bacterial infection markers and other immune mediators; and conversely (in the M2 state) act in an opposing manner and behave in an anti-inflammatory, healing and repair-focused state, triggered by mediators like Il-4 (Murray, 2017). When in the M1 state, macrophages act to sensitize nociceptors by releasing inflammatory cytokines including tumor necrosis factor alpha (TNFα), Il-1β, Il-6, growth factors, and lipids mediators such as prostaglandin E2 (Pinho-Ribeiro et al., 2017). Platelets are another component of the innate immune system known to induce nociceptive sensitivity through the release of pain causing factors (Ringkamp et al., 1994) triggering the release of ATP, Il-1β, and others that sensitize nerve terminals (Weth et al., 2015). Mast cells are activated during injury or stimulation of nociceptors, by vasoactive and proinflammatory neuropeptides Substance P and CGRP. Upon activation, mast cells release molecules including neuropeptides, histamine and other pain-causing mediators which proceed to further sensitize nociceptors (Gupta and Harvima, 2018). Importantly, in mouse models without mast cells, hypersensitivity does still develop indicating that multiple different immune cells are involved in this mechanism (Lopes et al., 2017). For example, in carrageenan-induced inflammatory pain models, neutrophils increase nociceptive sensitivity by producing cytokines and prostaglandin E2 (PGE2) (Cunha et al., 2008).

#### Adaptive Immunity

This role of the immune system in pain sensitization is not limited to the innate immune system, with the adaptive immune system also playing an important role in modulating nociceptive hypersensitivity. T cells including Th1 and proinflammatory Th17 subsets play a role in neuropathic pain by acting as a source of IFN-γ, chemokines, and IL-17A. IL-17A acts at nerve terminals to sensitize nociceptors by decreasing the rheobase (depolarization required to trigger an action potential) (Hu et al., 2007). The adaptive immune response also plays an important role in mediating local tissue damage and has been hypothesized to act as another source of mediators for the inflammatory soup. However, strong evidence suggests that T-cells do not have an essential role in pain sensitization in Complete Freund’s Adjuvant (CFA) induced hypersensitivity (Ghasemlou et al., 2015). B cells may also act as a source for components of the inflammatory soup and are able to secrete IgG antibodies that form IgG-immune complexes that can directly activate nociceptors (Jiang et al., 2017).

The interaction between nociceptors and immune cells is not limited to the site of injury as immune cells also interact with nociceptor cell bodies within the DRGs to produce pain, alter protein synthesis and induce sensitization (Kim and Moalem-Taylor, 2011) Many disease models show increased numbers of monocyte/macrophages, neutrophils, and T cells in the DRG (Hu et al., 2007), for example during nerve injury in rats, T cells are recruited and cause neuronal sensitization due to the serine protease inhibitor SerpinA3N inhibiting T cell-derived leukocyte elastase (Vicuña et al., 2015).

### Components of the Inflammatory Soup

The inflammatory mediators have a number of targets of action. Some of these molecules directly activate or modulate nociceptors, for example protons that directly enter terminals. Other molecules such as prostaglandins, bradykinin and ATP have their own receptors coupled to signaling cascades that enhance neuronal activity. Activation of these signaling cascades can cause modification of existing channels, an upregulation of trafficking of receptors or the downstream regulation of transcription.

#### Growth Factors and Cytokines

Growth factors such as NGF, are important regulators of neuronal survival during development (Ritter et al., 1991; Indo et al., 1996; Einarsdottir et al., 2004). However, importantly, in post-natal neurons the level of NGF regulates nociceptive sensitivity, and the increase in NGF derived from an unknown source of cells (Denk et al., 2017), causes an increase in nociceptive sensitivity (Lewin et al., 1993; Bennett et al., 1998) predominantly through the modulation of the capsaicin receptor TRPV1 (Chuang et al., 2001). Importantly, neutralizing this developmental process in the adult is a successful method for attenuating inflammatory pain in rodents and humans (Koltzenburg et al., 1999) and NGF antibodies represent an important new class of disease modifying drugs effective for the treatment of osteoarthritis (Lane et al., 2010).

Cytokines are small secreted proteins that include chemokines, interleukins, TNFα, colony stimulating factors, and interferons among others. Cytokines have a major established role in pain sensitization.

Pro-inflammatory cytokines, are a major class of secreted cytokines that include TNFα, IL-1, IL-17, IL-2, IL-4, and IFN also play a major role in nociceptive hypersensitivity. These molecules can have a major role in promoting hypersensitivity. This role is partly through activation of other local cells. For instance, TNF-alpha activity can induce IL-1β and NGF expression to increase pain sensitivity (Woolf et al., 1997). Primary afferents also maintain a repertoire of receptors for pro-inflammatory cytokines that have direct and indirect effects. For example, IL-1β signaling causes sensitization of sodium currents in primary afferents through p38-MAPK activation causing a reduction in the voltage dependent block of Nav1.8 and Nav1.9 (Binshtok et al., 2008).

Chemotactic cytokines, known as chemokines, have a particular established role in pain. By definition, in the immune system, these molecules have a chemotactic effect and a major part of their role in pain sensitization is recruitment of immune cells. However, many chemokines have receptors on sensory neurons that are directly activated GPCRs, able to modulate receptors and coupled to their own signaling cascades, for example CCL3 can sensitize TRPV1 currents (Zhang et al., 2005), and CCL5, CCL22, CXCL12 directly elicit calcium flux, in cultured neurons and cause pain hypersensitivity in rats (Oh et al., 2001).

#### Neuroactive Molecules

Neuroactive molecules including serotonin, derived from platelets, mast cells, and endothelium (Bardin, 2011), and histamine, derived from mast cells (Cenac et al., 2010), are other components of the inflammatory soup. The pain sensitizing action of histamine appears related to its ability to modulate TRPV1 sensitivity. For histamine, activation of Histamine Receptor H1 (HRH1) can drive nociceptive sensitization (Murray, 2017), through the activation of phospholipase C (PLC), a second messenger, to hydrolyze PIP2, that naturally antagonizes TRPV1. In a parallel pathway HRH1 activity through PLC can co-currently activate protein kinase C (PKC), which directly triggers TRPV1 sensitization through phosphorylation (Cao et al., 2013). Importantly, the HRH1 also acts via the TRPV1 channel to induce visceral hypersensitivity in patients with IBS and antagonists of HRH1 attenuate visceral nociceptive hypersensitivity *ex vivo*. Furthermore when IBS patients are treated with Ebastine, an antagonist for the H1 receptor, visceral neurons become significantly less sensitive, and in patients who had visceral hypersensitivity, their sensitivity reduced (Wouters et al., 2016).

#### Neuropeptides

Neuropeptides such as bradykinin and tachykinin are important nociceptor sensitizing agents released during tissue damage (Cesare and McNaughton, 1996). For instance, bradykinin release acts in two ways to sensitize neurons exposed to repeated noxious heat stimuli, firstly by releasing intracellular calcium by activating an inward current (Rang et al., 1991), depolarizing the nociceptor and generating action potentials, and secondly through activation of PKC and reducing the heat sensitive current threshold. PKC is regarded as a master regulator of both peripheral sensitization and central sensitization. PKC activation drives phosphorylation of a variety of target receptors and ion channels causing modulation of their activity. In particular PKC activity can drive receptor phosphorylation (Velázquez et al., 2007), it is also known to modulate cation flux through Nav1.9 and Nav1.8 sodium channels (Baker, 2005). Tachykinin peptides represent an important group of neuropeptides that are active in many physiological processes (Otsuka and Yoshioka, 1993). Tachykinins are key modulators of primary nociceptive afferents. Tachykinins control electrical excitability after repeated activation or injury. Tachykinin knockout mice (*Tac1* KO) show rapid adaptation in response to tonic mechanical stimuli and are not able to properly encode repetitive stimuli. Moreover, mechanical sensitization does not occur, hypersensitivity and paw edema were reduced after paw incisions (Gutierrez et al., 2019). Whilst Tachykinins have an established role in mammalian pain, drugging tachykinins has proved complicated. Neurokinin-1 receptor antagonists that prevent the actions of multiple pain related tachykinins such as substance P and neurokinin A have not been successful as analgesics in clinical trials despite their efficacy in preclinical models (Hill, 2000). Part of this failure may be due to lack of CNS penetration, and differences at the protein level in rodents. This also reflects the limitations of animal models which are affected by emotional aspects such as stress and also variations in compounds that affect stress such as NK1, potentially confounding analgesic affects.

#### Lipids

Another major class of inflammatory mediators are arachidonic acid and its lipid metabolites. A key group of lipid mediators are eicosanoids including the prostacyclins and the prostaglandins (PGE2), which induce nociceptor hypersensitivity. During inflammation the enzyme cyclo-oxygenase (COX), converts arachidonic acid to eicosanoids including PGE2 which increase nociceptive sensitivity, PGE2 sensitizes nociceptors partly through sensitizing TRPV1 responses through PKC and PKA activity (Sachs et al., 2009). There are two forms of the COX enzyme, COX1 and COX2. COX1 is constitutively expressed across the body however, it plays a major role in the regulation of gut physiology including mucosal protection, gastrointestinal secretion and motility (Hawkey, 2001). COX2 expression is induced by inflammation, and COX2 activity is the main contributor to eicosanoid-induced nociceptor hypersensitivity. Non-selective COX1/2 inhibitors are widely used for the treatment of inflammatory pain with varying efficacy and include ‘blockbuster’ drugs, indomethacin, ibuprofen, diclofenac and aspirin. Although, selective COX2 inhibitors were originally predicted to have a positive side-effect profile in reality the drugs showed a similar profile to existing drugs (Bresalier et al., 2005; Solomon et al., 2005; Nissen et al., 2016).

#### Non-host Factors

Non-host factors from bacteria, venoms, virus and parasites mediate sensitizing responses in nociceptive sensory neurons and may cause pain (Lau et al., 2019). This pain is partly the result of sensitization of nociceptors in reaction to the immune system, and partly triggered by the presence of pathogen derived molecules. For example, lipopolysaccaride (LPS) is a common surface molecule found on the membrane of Gram-negative bacteria and is a noxious by-product of bacterial lysis. LPS activates the Toll-like receptor 4 (TLR4) driving depolarization via the TRPV1 channel (Diogenes et al., 2011). In addition to TLR4/TRPV1 activation, LPS also acts directly on the TRPA1 channel independently of the TLR4 pathway (Meseguer et al., 2014). Moreover flagellin, bacterial toxins, and zymosan may also trigger detection of a pathogen by nociceptors and the resultant pain (Pinho-Ribeiro et al., 2017). The bacteria Staphylococcus aureus induces pain in mice independently of immune cell activity. Bacterial N-formylated peptides and the pore forming toxin α-hemolysin, induce calcium influx and action potentials in nociceptor neurons, inducing pain, and in the case of α-hemolysin most probably through direct pore formation (Chiu et al., 2013).

#### Peripheral Glia

Afferent associated glia play an important role in sensitizing responses. In part peripheral glia may act as damage sensors. CCL2 for instance induces the migration of macrophages, which signal to Schwann cells to maintain the infiltration of macrophages and ongoing allodynia (De Logu et al., 2017).

Recent evidence suggests that skin-resident peripheral glia, and in particular activation of specialized Schwann cells that act as mechanotransducers, are both necessary and sufficient for mechanical pain sensation (Abdo et al., 2019), however the potential role that these cells play in sensitization remains to be assessed. These cells appear to have a role in setting sensory thresholds and acute damage to these cells may therefore affect somatosensory thresholds, consequently targeting these cells has strong therapeutic potential. Given there seem to be particular cell types involved in nociceptive pain, the potential arises to target pain without impacting non-nociceptive modalities such as touch. These findings are reminiscent of Merkel cells that contain Piezo2 channels which are necessary for the encoding of tactile stimuli for A-beta fibers. Piezo2 positive merkel cells have been shown to be required for capsaicin mediated tactile hypersensitivity (Ikeda et al., 2014; Maksimovic et al., 2014). Together these studies suggest new therapeutic pathways to target specific cells involved in pain perception.

#### General Principles of Peripheral Sensitization

As alluded to these mechanisms function at a transcriptional and post-translational level to initiate pro-sensitization cascades that drive peripheral sensitization. Activation of p38 mitogen activated protein kinase by signaling cascades for instance is sufficient to further stimulate the transcriptional upregulation of TNF-α and IL-1β which then feedback to amplify the inflammatory response and maintain nociceptive sensitization.

## Central Sensitization

**FIGURE 2:**
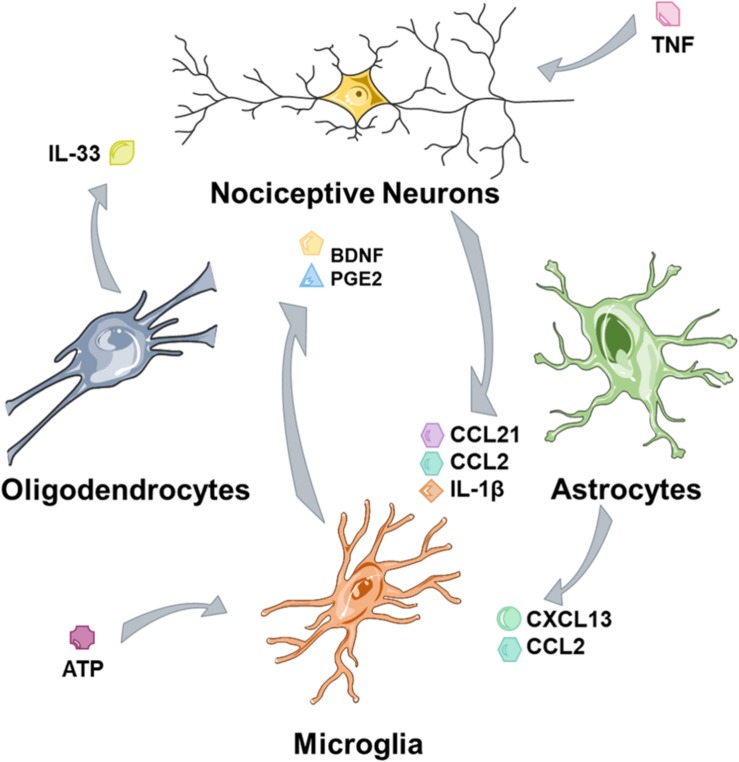
Glia and nociceptors are involved in cross talk mechanisms. Nociceptors, tissue damage and nociceptive neurons secrete factors that activate glia and trigger microglial activation. Microglia maintain nociceptive hypersensitivity through a range of mediators and astrocytes contribute further to this process. Oligodendrocytes may act as a further source of mediators.

Central sensitization refers to the increase in nociceptive sensitivity driven following injury at a central rather than a peripheral level. This is not narrowly defined, so some of these mechanisms also function at the level of the brain rather than solely at the level of the spinal cord (Latremoliere and Woolf, 2009). Moreover, these mechanisms function in many cell types (Figure 2), including neuronal, astrocytes, and microglia and to a lesser extent oligodendrocytes may also contribute.

### Glia

#### The Sequence of Glial Activation

Over recent years it has become increasingly apparent that multiple cell types are involved in the transition to chronic pain following injury. Some researchers have attempted to define the causes of these sequential responses. For example, ERK activation occurs in many spinal cord cells in a particular order. Using ERK phosphorylation as a surrogate for activation, it is apparent the first cells responding to nerve injury are neuronal and then microglia follow in the early (<2 days) phase, while astrocytes are activated last (Zhuang et al., 2005). It is clear that both microglia and astrocytes are both activated by nerve injuries and both populations show substantial morphological changes. This activation appears to be important for the maintenance of neuropathic pain (Zhuang et al., 2006). Oligodendrocytes also have a reported role in this transition however, the time frame and exact role have not been as well-characterized as the role of microglia and astrocytes.

#### Astrocytic

Astrocyte activation has been associated with mainly the late (maintenance) phases of neuropathic pain. Ablation of astrocytes using intrathecal astrocyte targeted toxins is able to reverse established neuropathic pain (>10 days in rats), although curiously this represents only a transient effect suggesting that inhibiting astrocytic activation alone is not a viable strategy for the alleviation of neuropathic pain. Moreover, spinal nerve ligations (SNLs), a model of neuropathic pain, induce the activation of cJUN in spinal astrocytes and inhibition of cJun activity is able to therapeutically reverse established neuropathic pain (Zhuang et al., 2006). Astrocytes can act as a source of activating chemotactic factors including CXCL13 and CCL2. CXCL13 appears to be mainly upregulated in astrocytes, and seems to be essential for neuropathic pain. There is evidence indicating that CCL2 and CXCL13 are acting through microglia (Jiang et al., 2016) Knockout of the cognate receptor for CXCL13, CXCR5, causes loss of SNL-induced hypersensitivity together with reduced microglial activation (Jiang et al., 2016). In common with other inciting factors from glia, it is not clear that astrocytes represent the major source for all of the inciting molecules. CCL2 is also expressed in injured neurons after nerve injury and it is unclear if CCL2 derived from astrocytes plays an important role in neuropathic pain since the source after nerve injury appears largely neuronal (Thacker et al., 2009). Given that astrocytes are mostly post-mitotic (Sofroniew and Vinters, 2010) and neuropathic pain in the post-injury state is maintained, it is possible that the role of astrocytes is to maintain continual microglial activation through microglial cell division.

#### Microglial Activation

Following tissue damage, inflammatory mediators are released into the spinal cord in much the same way that peripheral sensitization happens at the periphery. A key target and source of these inflammatory mediators is the microglia. In particular the spinal upregulation and activation of ionotropic, purinergic (ATP) receptors P2X7 and P2X4 (Tsuda et al., 2003) triggers an inflammatory cascade culminating in the release of brain derived neurotrophic factor (BDNF) (Ulmann et al., 2008) and eicosanoids such as PGE2. Damaged peripheral neurons are hypothesized to trigger this cascade through the secretion of inflammatory factors from vesicular release and through damage. Specifically, the damaging of a neuron triggers the trafficking of chemokine-containing vesicles and their release from neurons (de Jong et al., 2008). For example, the release of CCL21 from nociceptors (or intrathecal injection) triggers the upregulation of P2X4 receptors in microglia and blocking this upregulation in knockouts attenuates neuropathic pain (Biber et al., 2011). Moreover, the direct injection of these inflammatory mediators is sufficient to elicit a neuropathic-like condition. Alternatively, the early release of pro-inflammatory cytokines including TNF-alpha following tissue injury also activates pro-inflammatory pathways. For instance, TNF-α is able to activate the expression of matrix metalloproteinase 9 (MMP-9) in cultured DRG neurons. MMP-9 can then cleave IL-1β causing its releases and this in turn activates spinal microglia (Kawasaki et al., 2008).

The importance of microglia in chronic pain transition is now uncontroversial however, the exact mechanisms require further investigation to identify the druggable components of this response. Multiple groups have shown that inhibiting microglial function is sufficient for the prevention of the development of neuropathic pain although there are only limited effects on established neuropathic pain (Coull et al., 2005). Moreover, the delivery of microglia stimulated with ATP is sufficient to elicit temporary neuropathic pain like symptoms in rats, although the delivery of intrathecal stimulated microglia may not truly reflect neuropathic pain. Microglial BDNF expression is suggested to be responsible for this effect in male mice since conditional deletion in microglia (CX3CR1 positive cells) inhibits pain after nerve injury (Sorge et al., 2015), although, necessarily, these studies utilized tamoxifen which could have sex-dimorphic effects due to estrogen receptor targeting (Birzniece and Ho, 2015; Ceasrine et al., 2019); so more research will be essential to understand this sex-dimorphism. The microglia source of BDNF has good genetic (Sorge et al., 2015), pharmacological and immunolabeling support (Ramer et al., 2007) however it remains controversial. Embryonic fate mapping experiments and *in situ* hybridization have suggested that BDNF is not expressed in naïve spinal microglia (Dembo et al., 2018) and sequencing evidence suggests an absence of *Bdnf* transcripts in spinal microglia after partial nerve injury (Denk et al., 2016), so repeating the lineage tracing experiments in the context of nerve injury will be essential for resolution. Together, microglia play an important role in increasing nociceptive sensitivity by providing pro-inflammatory factors, functioning similarly to peripheral immune cells, although their exact roles remain unclear and the pathways are not convincingly established.

#### Oligodendrocytes

Oligodendrocytes may also have a role in this transition to neuropathic pain. There is conflicting evidence that oligodendrocytes may be both pro-inflammatory and anti-inflammatory. Ablation of oligodendrocytes can trigger a central pain syndrome (Gritsch et al., 2014). Other reports indicate that the production of IL-33 may cause activation of astrocytes and microglia, although the factors that trigger oligodendrocyte changes remain unresolved (Shi et al., 2016). Similarly, to the issues we describe in microglia, it is complex to ascribe the function of IL-33 to oligodendrocytes since a major source of IL-33 is astrocytes (Vainchtein et al., 2018). Whilst these studies show oligodendrocytes may be able to modulate pain, the mechanistic evidence for their role remains highly limited and the directionality remains controversial.

### Central Spinal Circuit Potentiation

High frequency activity at nociceptive connections in the spinal cord can cause mechanisms akin to long term potentiation (LTP) to occur wherein there is synaptic strengthening and a lower threshold is required to elicit a depolarization (Woolf, 1983). Similarly to LTP (Bliss and Lomo, 1973), there are several potential mechanisms all of which converge on increased intracellular calcium. In the same way as central sensitization, the key mechanism is NMDA (*N*-methyl-D-aspartate) receptor opening. The NMDA receptor is blocked at rest by a magnesium ion in its pore (Mayer et al., 1984). The coincidence of glycine (or serine or alanine), glutamate and strong voltage depolarization removes the magnesium block to the pore. The NMDA receptor shows high selectivity for calcium and the rise in intracellular calcium triggers cytosolic protein kinases which modulate receptor activity and increased excitability, in particular causing synaptic strengthening partly by increasing the AMPA (α-amino-3-hydroxy-5-methyl-4-isoxazolepropionic acid) receptors at the synaptic cleft through increased trafficking to the plasma membrane, receptor phosphorylation (Zou et al., 2000) and partly through a calcium-dependent transcriptional cascade. The most general mechanism of LTP holds true in dorsal horn neurons undergoing heavy stimulation and repetitive stimulation is known to cause synaptic strengthening (Ji et al., 2003). However, there are some critical differences to classical hippocampal LTP, for instance they do not drive the same transcriptional cascades and some signaling molecules such as p38-MAPK follow a different pattern, wherein inhibition supports LTP (Kelly et al., 2003) but p38 increases in some pain sensitization states (Crown et al., 2008). Moreover classical LTP only causes synaptic strengthening, whilst in dorsal horn neurons undergoing central sensitization there are a wide variety of neuronal responses (Ji et al., 2003) suggesting that classical LTP does not completely explain neuropathic pain. Regardless of the differences between LTP and its spinal correlate, central circuit potentiation theories have had a fundamental impact on the way that we treat post-operative pain (Wall, 1988; Woolf and Chong, 1993) and encouraged the development of blocking analgesic approaches to post-operative pain relief. Mechanisms of central sensitization are interlinked, and disinhibition for instance (the loss of inhibition in neuropathic pain) is directly linked to mechanisms of central sensitization through coupling proteins such as STEP61, that promote NMDA subunit (GluN2B) phosphorylation during loss of inhibition.

### Disinhibition

**FIGURE 3:**
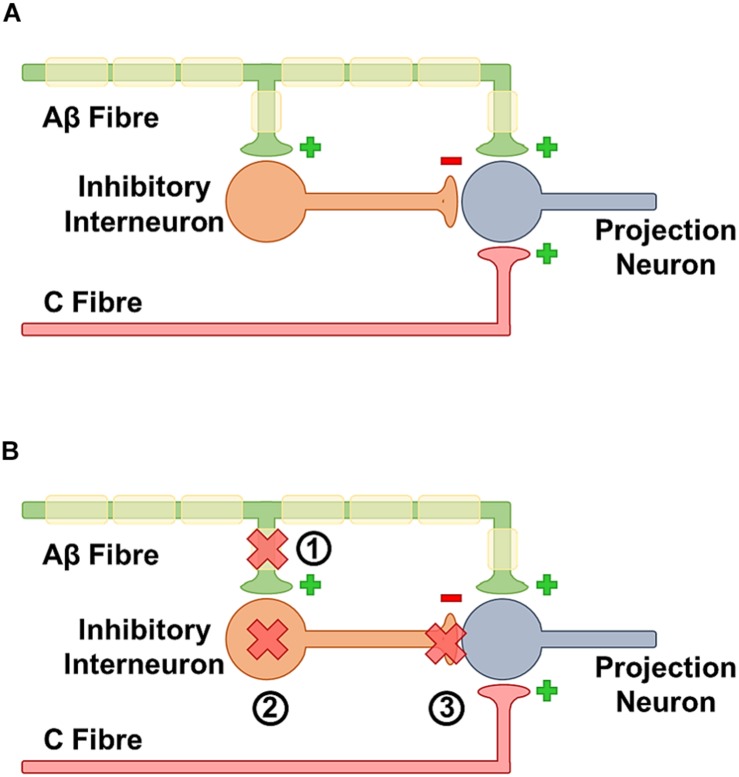
**(A)** In the healthy state insufficient activation by the C fibers is unable to trigger neurotransmission because they are gated by activation of inhibitory neurons by A beta fibers. Together this means that a beta activation inhibits pain transmission. **(B)** In the sensitized state, activation of a beta fibers is unable to block c-fiber response. There are multiple contributing mechanisms for this phenomena. **(1)** After nerve damage there is an overall loss of afferent fibers, some of these fibers feed onto inhibitory neurons so the loss may decrease activation. **(2)** Inhibitory interneurons can be lost at a whole level. **(3)** The GABAergic synapse may be less efficient if there is a change in the ability of GABA to hyperpolarize cells.

The gate control theory of Wall and Melzack posited that certain kinds of chronic pain were triggered by a loss of a central gate for pain (Melzack and Wall, 1965). The idea of this gate was that non-painful stimuli may activate nociceptors but not be able to overcome this gate. A proposed structure is that A-Beta touch fibers fire and activate interneurons which then inhibit firing of second order projection neurons. Conversely when there is sufficient nociceptive c-fiber firing, the gate is overcome and pain is transmitted through projection neurons. They thought this in part because rubbing can relieve pain suggesting integration between touch and pain and that A-beta touch fibers may inhibit c-fibers (Figure 3) Moreover, during allodynia non-painful stimuli become painful, and finally lesions of the system do not relieve neuropathic pain suggesting a major central component. There is significant experimental evidence that the activity of low threshold mechanoreceptors is able to gate nociceptors and that increasing nociceptor activity is necessary to overcome the gate. Experimentally, activation of a-fiber nociceptors with channel rhodopsin can cause pain and this can be inhibited by the co-activation of low threshold mechanoceptors (Arcourt et al., 2017). A proposed identity of the classic gate is the inhibitory GABAergic and glycinergic neurons of the dorsal horn. In neuropathic pain conditions, central disinhibition represents a potentially important pathological mechanism. Specifically, electrophysiological recordings have identified that there is a decrease in the inhibitory post-synaptic potential (Moore et al., 2002) suggesting a loss of inhibitory tone. More recent work in *Drosophila* shows that this loss of inhibition is a strongly conserved mechanism essential for at least some forms of nerve injury associated hypersensitivity (Khuong et al., 2019a, b). Moreover, the specific ablation of spinal inhibitory interneurons seems sufficient to cause neuropathic pain reinforcing that dorsal horn inhibitory interneurons can act as pain gates (Foster et al., 2015; Petitjean et al., 2015). A number of mechanisms have been suggested that could explain the observed decrease in central inhibition.

#### Anatomical Loss of GABAergic Neurons

One of the first theories to explain loss of inhibition was anatomical loss of GABAergic neurons in the spinal cord (Moore et al., 2002). Following nerve injury by CCI and SNI, firstly in the rat but also in mice, some groups have observed a decrease in the numbers of spinal neurons (Inquimbert et al., 2018). The mechanism for this is hypothesized to be primarily excitotoxic, and some groups have described a rescue of these phenotypes with both caspase inhibition or anti-oxidant treatments (Scholz et al., 2005; Yowtak et al., 2013) However, these hypotheses remain controversial. Multiple detailed stereological anatomical studies have suggested there is no loss of GABAergic neurons following nerve injuries, and two independent groups have suggested tactile allodynia occurs in the absence of a decrease in GABAergic neurons (Polgár et al., 2003, 2005; Polgár and Todd, 2008; Leitner et al., 2013). Moreover, the estimates from the groups who report dorsal horn cell death vary very widely, from as little as 10% in lamina I-II of the dorsal horn to as much as 25% of total neurons in the dorsal horn (Scholz et al., 2005; Inquimbert et al., 2018). Finally, the dying cells have not been reliably identified as neurons and other groups suggest that the apoptotic profiles are co-localized with microglial markers (Polgár et al., 2005). Therefore, the relative contribution of GABAergic cell death to neuropathic pain remains unresolved. Whilst some evidence exists, the evidence of the contribution is contradictory and conflicting, and further investigation with more precise techniques is in order.

#### Loss of Peripheral Input

An additional theory explaining these observations is that following a loss of peripheral afferents there is a loss of activity to inhibitory neurons and in a feed-forward manner this causes the loss of inhibition since there is less primary afferent input (Leitner et al., 2013). Given that there are fewer primary afferents of all classes there are less feed-forward inputs to inhibitory interneurons as such this can cause a decrease in activity and explain the decrease in inhibitory post-synaptic potentials. And indeed, spinal recordings do show a reduction of spinal inhibitory post-synaptic potential in the interneurons connecting to projection neurons when stimulated from mechanosensitive A-beta fibers following nerve injury (Lu et al., 2013). Thus there is evidence that a loss of peripheral input upon central inhibitor neurons can underlie some of the phenotype of reduced pain sensitivity however, this evidence remains limited to only a few pain conditions and again future studies are warranted.

#### Changing Role of GABA and the Regulation of KCC2

The inhibitory nature of GABA receptor activation itself is also proposed to shift during the transition to a neuropathic pain state more similar to the role of GABA in the developing brain. Due to the chloride balance of the early brain, GABA has a divergent depolarizing rather than hyperpolarizing activity in this context. In such circumstances, GABA effectively acts as an excitatory neurotransmitter (Ben-Ari, 2002) and in early development actually represents the primary excitatory neurotransmitter (Leinekugel et al., 1999). Part of the reason for this appears to be that BDNF stimulates a transcriptional pathway culminating in the decreased expression of KCC2, a potassium chloride co-transporter (which under normal conditions will extrude chloride ions), thus enhancing the intracellular concentration of chloride ions. This shifts the intracellular balance of chloride ions and makes GABA excitatory. This pathway is also proposed to have a role in the development of neuropathic pain given that BDNF is released following nerve injury from microglial activation cascades (Coull et al., 2005). Since, the primary role of GABA in these circuits is thought to be analgesic (or more precisely gating stimuli), this changing role causes hypersensitivity. This mechanism is not limited to rodent models and KCC2 mediated disinhibition is observed in human models (Dedek et al., 2019). Finally, targeting this pathway with KCC2 activating small molecules is an effective and fairly specific method for analgesia in rodents (Gagnon et al., 2013). However, given that increasing GABAergic inhibition causes a general suppression of pain in neuropathic pain models (Hwang and Yaksh, 1997; Eaton et al., 1999; Zhang et al., 2011; Bráz et al., 2012; Petitjean et al., 2015; Fandel et al., 2016) as well as patients (Zuniga et al., 2000; Harmer and Larson, 2002) the exact details of this potential mechanism remain somewhat unclear, since the observations of GABA induced analgesia are inconsistent with a complete reversal of GABAergic function. It is therefore more likely that in most cases this represents only a contributory phenomenon, whereby hyperpolarizing currents are attenuated in response to GABA in a small subsets of neurons, rather than a complete current reversal underlying neuropathic pain as originally claimed. Recent studies in the hippocampus suggest that KCC2 downregulation may only have a limited impact on GABAergic signaling. The downregulation of potassium leak conductance may actually underlie the observations of increased excitability following nerve injury potentially unifying these apparently dichotomous results. Whilst the intracellular change in chloride does result in depolarization of the reversal potential of chloride (the electrochemical potential of the membrane at which there is no motive force on ions), the change in the reversal potential is almost entirely compensated by an increase in the resting membrane potential limiting the impact on GABAergic signaling. Loss of KCC2 downregulates the membrane expression of Task-3 in the hippocampus and this downregulation appears to enhance the coupling between excitatory inputs, importantly this is independent of GABAergic signaling (Goutierre et al., 2019). KCC2 downregulation has an important role in neuropathic pain and targeting KCC2 appears to have a good analgesic efficacy in preclinical models and importantly changes in KCC2 mediated inhibition are conserved to human models (Dedek et al., 2019).

#### A Decline in Inhibitory Neurotransmitter Synthesis

A decline in GABA levels in the spinal cord and brain may underlie some aspects of neuropathic pain and changes in the GABAergic synthesis system have been identified in a wide variety of models at both central and spinal levels. In the nucleus raphe magnus (NRM), following Nerve injury and CFA injection in Rats, there is a decline in *Glutamic acid decarboxylase* (*Gad) 2*, and reversing this using Histone deacetylase (HDAC) inhibition relieves aspects of neuropathic pain (Zhang et al., 2011). This represents a potential supraspinal site of regulation of pain, since critical descending analgesic circuits are located here. Whilst there are clear differences at supraspinal sites, other studies have also examined GABA at a spinal level. Many studies have examined the loss of GABA machinery at the transcriptional level; it is clear that *Gad2* (encoding for GAD65) transcripts are decreased in the spine although it is unclear whether this change is driven by transcriptional downregulation or a loss of neurons (Bráz et al., 2012). There is no clear difference in GAD67 levels potentially arguing against a general loss of GABA neurons, since GABAergic neurons express both forms. However, these effects could also be a result of negative feedback mechanisms that maintain GAD67. Other studies have measured the concentration of amino acids in the spinal dorsal horn by high performance liquid chromatography (HPLC) and electron microscopy, these studies have found somewhat contradictory results. Some investigators suggest a substantial decline in GABA concentration in the dorsal horn (Liu et al., 2004) in central pain (from spinal injury), and peripheral nerve injuries (Shen et al., 2014) however, others have detected no significant changes by immunolabeling and electron microscopy (Polgár and Todd, 2008). Moreover, many of these studies do not address the mechanism of changes in GABA synthesis since the majority of these studies have not been able to determine the etiology of loss of GABA. Therefore, the relative role of loss of GABA synthesis remains somewhat unclear.

#### Loss of Central Inhibition in Human Systems

The system shows additional complexity as the reduction in GABA is not limited to the spinal cord. At least in neuropathic human patients there is a loss of central GABA using quantitative magnetic resonance spectroscopy, a method by which an MRI can resolve the molecular composition by examining the amino acid concentrations of a specific space. The only study so far to utilize this technique showed that there was a significant decrease in the levels of GABA in the contralateral thalamus (the contralateral thalamus is innervated by the ipsilateral side). This suggests multiple levels of regulation of pain transmission including multiple levels of inhibition (Henderson et al., 2013). The utility of this technique has been somewhat limited by the fact that it is technically complex to image at the spinal cord level and this has meant it is not possible to determine whether there is a loss of GABA at the spinal level in human patients. Whilst, it is unclear if GABAergic signaling is defective in the spinal cord of human patients, administration of GABA receptor agonist baclofen spinally through intrathecal injection can both cause some relief in cases of neuropathic pain amongst human patients as well as in pre-clinical models (Hwang and Yaksh, 1997; Zuniga et al., 2000; Harmer and Larson, 2002). However, it is difficult to isolate the effects of the GABA receptor agonists on brain regions rather than acting locally in the spinal cord since the route of administration (intrathecal) provides no certainty of spinal specificity. Other drugs which are known to target GABAergic pathways are also used in treatment refractory neuropathic pain patients including GABA reuptake inhibitors (Todorov et al., 2005), and GABA transaminase inhibitors including valproic acid (Gill et al., 2011). However, many of these drugs are regarded to be promiscuous and target many pathways making it difficult to identify the specific mechanism of action. Moreover, many of these drugs are general off-label indications with limited clinical trial support (Gill et al., 2011). All of these drugs also necessarily act centrally; the central action of GABA modulating drugs means the assignment of these drugs to any spinal mechanism cannot be reliably assessed since modulating inhibition in the brain could also explain analgesia and the separation of these functions is non-trivial. Together, there is evidence of a reduction of inhibitory tone in humans and reinforcing central inhibitory tone through treatments that increase levels of inhibitory spinal transmitters may relieve neuropathic pain, although the level at which these treatments functions remains unclear and their exact mechanism is not well-understood.

## Current Issues in the Treatment of Pain

**FIGURE 4:**
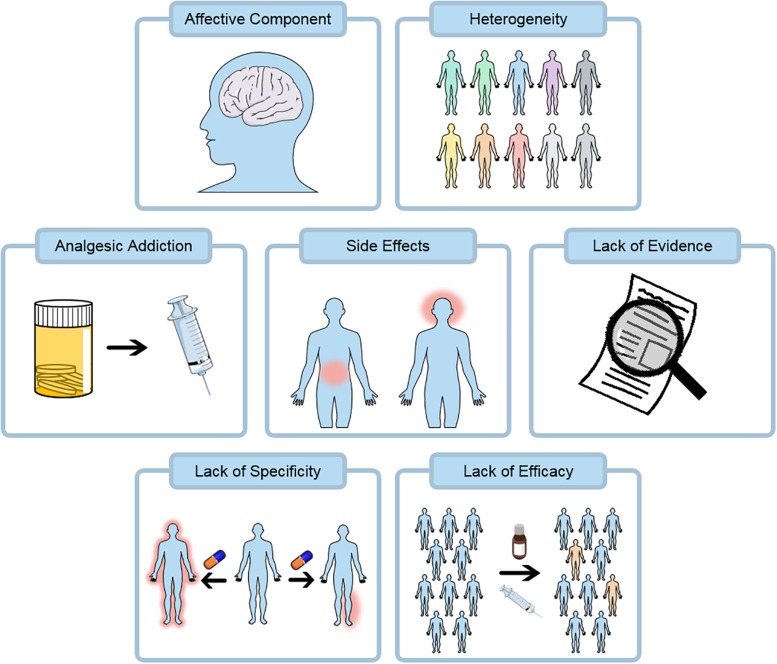
Pain therapies at present have a number of challenges. Opioids remain a key class of analgesics and are heavily associated with addiction, this property may be shared by many pain killers including the gabapentinoids. Current and emerging treatments are both associated with substantial side effects including gastrointestinal and central effects. Many existing treatments do not have a substantial evidence base supporting their use from robust randomized clinical trials. Pain relief does not act in a specific localized manner at present and side-effects are partly due to this. Finally, many pain treatments lack substantial efficacy and the number needed to treat is often greater than 5.

Superficially alleviating pain is obvious (Figure 4), treating the underlying condition removes inciting factors underlying the pain state and is expected to alleviate the pain. However, in some cases relieving the underlying cause of pain does not relieve the pain. Moreover, in many cases there is no clear etiology or targetable biological cause for the pain. A range of medications and interventions have been developed to address this problem however, they provide limited relief and cause their own problems.

### Addictive Properties of Analgesics

Addiction due to pain relief now represents a major crisis in medicine. Since the early 1990s, opioids have played a much more important role in treating pain in the community, with devastating results (Makary et al., 2017; Curtis et al., 2019; Glare et al., 2019). Opioid addiction is a severely disabling condition and is associated with a high disease morbidity and devastating social results (reviewed in depth by Volkow et al., 2019). The rise in opioid treatment has led to a public health crisis, driven primarily by prescription drugs, causing a fivefold increase in the rate of overdoses since 1999 (Centers for Disease Control and Prevention [CDC], 2013). Opioids are not positive disease modifying drugs for pain and cause tolerance and hyperalgesia. The opioid epidemic has partly been driven by a failure in education; for instance many clinicians were misled by data suggesting addiction under normal brief use is rare (Porter and Hershel, 1980), partly this has been a result of extensive and misleading marketing campaigns by elements of the pharmaceutical industry for inappropriate indications (Meier, 2007; Van Zee, 2009; Leung et al., 2017). Recent, high quality evidence suggest that opioids cause substantial addiction under regular use and as many as one in 16 surgical patients become opioid addicted (Volkow and McLellan, 2016; Leung et al., 2017). Furthermore, longer term treatment with opioid drugs can induce hyperalgesia creating a cycle of heightened pain responses necessitating greater doses; subsequently, these drugs are no longer recommended (as first or second line treatments) for neuropathic pain (Finnerup et al., 2015). Finally, clinical trial evidence suggests the efficacy of opioids is actually somewhat limited (corresponding to an improvement of around 0.67 cm on a 10 cm pain scale). Whilst other analgesics such as gabapentinoids were initially suggested to not have addictive properties, there is increasing evidence that these drugs are also addictive (Chiappini and Schifano, 2016). This highlights the importance of increasing analgesic specificity to avoid addiction.

### Evidence for Existing Long-Term Treatments

Many current methods for relieving pain diseases in the long-term, such as low back pain, lack a solid evidence base from randomized clinical trials (Fairbank et al., 2005). In particular there is no high-quality evidence that patients benefit from many of the surgical procedures prescribed to treat chronic pain such as dorsal rhizotomies, spinal fusions and arthroscopies. Orthopedics is based on the assumption that correcting tissue anatomy will resolve pain. In fact in many cases a placebo surgery may be just as effective and overall cure rates remain low from these procedures (Moseley et al., 2002; Fairbank et al., 2005), additionally these treatments can cause serious complications that should not be ignored. Moreover, the recent development of unregulated stem cell treatments including stromal derived material is concerning, as these therapies have not demonstrated efficacy through rigorous clinical trials (Turner, 2015). Ongoing pain makes patients vulnerable and patients require protection from procedures with poor evidence of efficacy.

### Limited Efficacy of Long-Term Treatments

Whilst the relief of nerve compression can promote long-term relief in certain cases of trigeminal neuralgia, where there is microvascular compression of the trigeminal nerve, in general this kind of surgical approach is unsuitable for most forms of neuropathy which lack an obvious mechanical etiology. A number of more recent approaches have been created in an attempt to address the lack of long-term therapies. The most obvious local therapies for pain that show considerable efficacy in small animal models are nociceptive ablation using high doses of specific agonists and blocking transmission, for example using local anesthetic injections. However, the use of non-specific local anesthetics is associated with considerable side-effects since they are not selective to particular voltage gated sodium channels and nociceptor ablation using capsaicin creams shows only mild efficacy in the clinic (Finnerup et al., 2015). Finally the analgesic themselves retain often limited efficacy, for the most effective drugs with good evidence the number needed to treat (number of patients treated for one patient to receive > 50% analgesia) is 3.6 for tricyclic antidepressants and > 5 for pregabalin, gabapentin and serotonin noradrenaline reuptake inhibitors (Finnerup et al., 2015).

### Specificity of Existing Pain Relief

Current pain relief works in a non-specific manner, In general current treatments for neuropathic pain focus on the block of signaling pathways (calcium and sodium channels) and the enhancement of descending control (for example opioids). For instance Gabapentinoids are largely calcium channel blockers originally developed for the treatment of epilepsy, conversely carbamazepine, an effective drug for trigeminal neuralgia blocks sodium channels. There are clear issues with this strategy. In general the nervous system uses conserved pathways for activity, whilst this means that drugs targeting other diseases, in particular epilepsy, are available, this also contributes to the broad array of side effects (Gilron et al., 2005; Derry et al., 2015; Mathieson et al., 2017). Many of these medications affect multiple neural systems, and cause side effects most importantly perhaps being fatigue. The limitations are not limited to drugs that act on the nervous system, for example selective COX-2 inhibitors Celecoxib and Rofecoxib cause severe thrombotic cardiovascular side-effects driven by their effects on non-target tissues (Bresalier et al., 2005; Solomon et al., 2005). This is highly problematic given target populations for these medications often have cardiovascular co-morbidities. Together the side effects impose significant limitations on the dose and efficacy of existing pain medications. Beyond the lack of efficacy and dose, these side effects impose a significant burden on quality of life in patients undergoing long term treatment. Even in cases where the pain is relieved, these side effects cause significant functional disability.

### Affective and Psychological Components

Chronic pain and related conditions such as addiction impose a significant affective disease on people. Existing pain treatments do not adequately address the burden of living with pain in part because they do not address the affective component. Chronic pain often co-occurs with serious psychiatric diseases such as depression and anxiety. There is evidence that chronic pain may directly contributes to these conditions and acute pain causes depressive symptoms in mice through a central mechanism (Sieberg et al., 2018; Zhou et al., 2019). Pre-existing depression may also predispose an individual to pain and additionally depression’s manifestation frequently involves somatic symptoms (Simon et al., 1999). Current targets may not be adequately addressing these central pathologies and these factors are not typically addressed by current behavioral models. Psychological factors also affect the intensity of the pain that is experienced, with catastrophization representing markers for pain intensity that is experienced (Sullivan et al., 1998; Tripp et al., 2009; Ogunlana et al., 2015). It is important to reflect whether our existing strategies actually address these aspects of pain pathology and if not how these could be better modeled. Addressing pain through for example enhanced spousal support for instance can have a substantial positive benefit on quality of life and these non-pharmacological interventions should not be ignored (Ginting et al., 2010).

Existing treatments and some emerging therapies retain limitations in their ability to promote functional recovery. Firstly, the aforementioned pain comorbidities can make functional living difficult, including maintaining employment and additional costs related to healthcare are associated with residual pain. Beyond this, not effectively treating pain can be dangerous. There is evidence that patients tend to self-medicate, often with harmful consequences such as high alcohol intake or the use of drugs (Alford et al., 2016). Withdrawal effects of drugs and alcohol can cause hyperalgesia and this contributes to the pathology and co-morbid addiction further adds to the societal and personal harms of pain (Egli et al., 2012). Not adequately addressing pain risks is exacerbating these effects and is the result of inadequate pain control. Given that these associated affective and psychological components are a major element of pain pathology, it is important that they are better addressed through new therapies and that therapeutic combinations with psychological treatments could be more frequently utilized.

### Pain Heterogeneity

At least part of the difficulty in treating pain is the biological heterogeneity of painful disorders. Superficially similar disorders such as neuropathic pain can have varying treatment efficacies that are even dependent on location of pain. For instance, trigeminal neuralgia, a neuropathic pain subtype specifically affecting the trigeminal nerve is unique in its strong therapeutic response to carbamazepine (Wiffen et al., 2014). Stratifying pain patients by symptomology already has utility, for instance in differentiating inflammatory and neuropathic pain. However, more detailed molecular analyses now suggest substantial heterogeneity in the pathologies underlying pain even with apparently similar symptomology. For instance, in the biologically heterogenous bladder pain syndrome, elements of the disease mechanism can be identified by finding clusters of patients and key genes that are responsible for these phenotypes such as the identification of CCL21 and FGF8 in bladder sensitization (Offiah et al., 2016). Similarly IBS-C (IBS with constipation), a subform of IBS typified by constipation, shows specific regulation of 5-Oxo-eicosatetraenoic acid (5-OxoETE), a polyunsaturated fatty acid in colonic biopsies (Bautzova et al., 2018). 5-OxoETE triggers sensitization and these results suggest that IBS can be sub-classified to reveal specific disease targets. Considering their different etiologies, targeting different aspects of the pathway may be more effective in some instances for relief than common elements with the caveat that this may substantially increase treatment costs. However, if subtypes classify treatment responses to existing medications this may have substantial and immediate therapeutic potential. In order to better classify pain diseases, we will need better biomarkers. Some promising approaches have been put forward in this field. For instance proteomic studies of the cerebrospinal fluid from pain patients have identified new potential biomarkers for complex diseases such as fibromyalgia (Khoonsari et al., 2019), studies like this offer substantial potential as sources for both the discovery of disease mechanisms and biomarkers. Similarly in neuropathic pain, deeper datasets characterizing the changes that occur in biopsies, quantitative sensory testing (to identify defective modalities), proteomic and genetic screening strategies may be able to identify subtypes of neuropathic illness (Themistocleous et al., 2018). There will be substantial promise in using these emergent methods to classify and provide more personalized pain treatments that are stratified by the underlying mechanism of disease.

## Developing Long Term and Local Therapies for Pain

**FIGURE 5:**
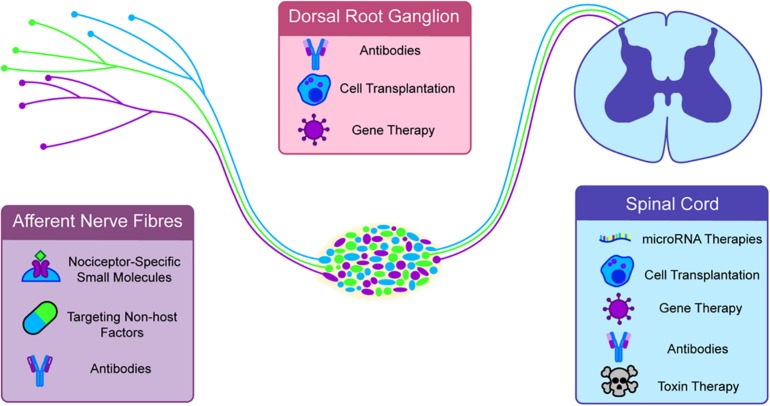
Novel and emerging therapies are generally targeted to specific parts of the nociceptive circuit. Antibodies, specific small molecules and non-host factors can be targeted at afferent nerve endings. The dorsal root ganglia (DRG) can be targeted with Gene and cell therapies. A major growing target for therapy is the spinal cord. In nociceptive sensitization this appears to be a major site of hypersensitivity and targeted toxins, cell transplants, gene therapies, micro-RNA therapies, and antibodies have all shown some efficacy in long term pain.

Whilst there are many available treatments for the short-term relief of pain, it has proved difficult to relieve pain in the longer term in any mammalian system without substantial side effects. This is particularly the case for neuropathic pain. A key reason for this is that current available therapies for neuropathic pain focus on the block of peripheral or central neurotransmission rather than altering factors underlying the maintenance of pain such as glial activation, central sensitization and loss of inhibition. Furthermore, all systemically acting drugs have potential for widespread side effects given their lack of specificity for neurons in the pain pathway. Historical approaches to the problem of intractable chronic neuropathic pain have been largely surgical, and the majority of these approaches revealed a singular truth, simply lesioning parts of the nervous system does not create pain relief in the long-term and many of these procedures had considerable adverse effects (Melzack and Wall, 1965). The expansion of molecular biological methods in pain research have driven further expansion in the potential pathways for treatment. Here, we explore how these advances have paved the way for new treatments (Figure 5).

### Genetic Therapies

Gene therapies have been increasingly proposed to treat intractable pain. Early methods involved the gene transfer using GAD65 using herpes simplex viral vectors to enhance inhibitory tone (Liu et al., 2004). More recent efforts have used the delivery of adeno-associated virus (AAV) containing glutamate activated chloride channels to peripheral neurons to promote long lasting pain relief by increasing hyperpolarizing GABA currents after ivermectin (designer drug) administration (Weir et al., 2017). Not all of these strategies will necessarily target neurons, delivery of anti-inflammatory cytokine IL-10 using lentiviral mediated transfer has also shown promise for neuropathic pain (He et al., 2013). Another recent development is the targeting of novel miRNA pathways; there is growing evidence for the involvement of miRNAs in both the maintenance and in initiation of chronic pain. Synthesized single strand inhibitors of these miRNAs have recently shown prolonged efficacy of weeks (Tramullas et al., 2018). An advantage of these methodologies is they show long lasting relief and, in some cases, may provide local relief targeting pain specific pathways or using drugs unable to cross the blood–brain barrier. Whilst some miRNAs have a role in maintenance of pain, overexpression of other miRNAs may have a role in promoting resolution most likely through modulation of chemokines. A considerable disadvantage of genetic therapies for pain is that we do not yet understand the consequences of long-term inhibition of these pathways, moreover, the route to market for genetic therapies is complex and there remain doubts as to the safety of viral vectors for genetic engineering. Whilst AAVs for instance have been safely used in many clinical trials (Seymour and Thrasher, 2012), there is good evidence that AAV integrations can cause hepatocellular carcinoma in mice (Donsante et al., 2007; Chandler et al., 2015) and recent evidence shows that novel CRISPR gene targeting methods may increase the risk of AAV integration (Nelson et al., 2019) and this could also be genotoxic. Furthermore, host responses to viral vectors have not yet been fully addressed in the spinal cord or dorsal root ganglia (DRG) and it is conceivable that these immunogenic responses could have long term negative effects. Moreover, the generation of targeting methods to affect specific cell types of interest may improve efficacy and tolerability, although without specific targeting, options are limited to vectors that have few negative effects in other local cell types. Together genetic therapies offer considerable promise for pain however, the limitations affecting much of the gene therapy field impose similar limits in pain therapeutics.

### Cellular Therapies

As in other fields some researchers have proposed and developed cell transplants to address pain. These cell transplants have focused on two major therapeutic targets, enhancing central inhibition by increasing spinal GABAergic signaling and inhibiting inflammation. Early work in pain cellular therapies utilized genetically modified cancer cell lines that were designed to act as GABA pumps however, the source of cells is not considered suitable for therapeutic purposes (Eaton et al., 1999). Later work suggested that it was possible to implant precursors from mouse fetal forebrain (the medial ganglionic eminence) of GABAergic neurons into mouse spinal cords and that with this method it was possible to relieve neuropathic pain for weeks from a spared nerve injury (Bráz et al., 2012) and chemotherapy induced neuropathy (Bráz et al., 2015). These cells differentiated largely into GABAergic neurons however there are important caveats, namely that these cells were derived from embryonic mice which is not a suitable source of cells for human transplants, as mouse cells cannot be used in humans without significant genetic modification. Moreover whilst there is a large bias toward GABAergic specification, (30%) other cell types including astrocytes were present in the grafts (Bráz et al., 2012, 2015; Etlin et al., 2016). Following the success of this work another group was able to create mouse forebrain-like progenitor cells from human embryonic stem cells and implant these into the injured spinal cord. These cells survived (in immunodeficient hosts) and this transplantation was able to reverse some of the side-effects of spinal cord injury specifically, heat hyperalgesia, mechanical allodynia and bladder dysfunction in long term studies (∼6 months therapy) (Fandel et al., 2016). However, these studies together retain substantial limitations; in particular, we do not fully understand the potential consequences on sensory-motor function of increasing the number of inhibitory neurons in the spinal cord or whether long term spinal cord reorganization could become pathological. Moreover, it is not clear if the transplantation of progenitor cells which are proliferating may have negative effects. Further studies will be essential to identify whether synaptic reorganization is a limiting factor for cell transplants targeting these pathways. There is substantial promise for these therapies to be adapted for iPSC, creating the possibility of autologous cell therapies. An alternative promising cellular approach is inhibiting inflammation using bone marrow-derived stromal cells (BMSCs) delivered intrathecally (Chen et al., 2015). In contrast to GABAergic transplants, these cells act as a source of pain killing mediators, in particular, releasing TGF-β1. These cells can home in on injured DRGs and only small numbers appear to be required for analgesia. Whilst these cells show considerable potential, their survival and potentially their efficacy appears limited to ∼70 days, so it remains unclear if these are a viable pain therapeutic at present. There are also general limitations to this approach, *in vitro* expansion of mesenchymal stem cells appears to cause an increase in their immunogenicity and autologous mesenchymal-type stem cells including BMSCs may be subject to NK-cell mediated lysis *in vivo* (Sotiropoulou et al., 2006; Spaggiari, 2006). Furthermore, these experiments used rodent MSCs and there is no evidence that human cells have the same effect. Together these therapies show substantial promise although their long-term safety, tolerability and efficacy remains unclear.

### Antibodies and Targeted Therapies

Antibodies are now a significant group of therapeutics in immunology (Chan and Carter, 2010) and a growing class of therapeutics for pain. Modulating the immune system has therapeutic potential for pain by resolving inflammation and moreover a number of pain specific therapies have been developed in recent years. NGF represents a major drug target for pain. Therapeutic antibodies have been developed that target NGF since it is a critical component of inflammatory pain (Denk et al., 2017). These antibodies can show considerable efficacy however, they are also associated with side effects. In particular, NGF neutralization can trigger neuropathy through a loss of trophic support (Einarsdottir et al., 2004; Testa et al., 2018). Moreover, a complete loss of pain perception is not necessarily helpful and treatment with anti-NGF medications can cause an increase in osteoarthritis pathology that caused an initial halting of anti-NGF therapies (Lane et al., 2010). This is thought to be partly driven through a loss of protective pain sensation. Additionally, these treatments at higher doses cause sensory abnormalities in many patients. More recent trials have not suggested a high frequency of these adverse events so it remains hopeful that anti-NGF therapies will become effective clinical tools for osteoarthritis. Similarly anti-CGRP antibodies have substantial promise for migraine therapy (reviewed in depth by Goadsby et al., 2017 and Silberstein et al., 2017). Alternatively, similar antibody strategies have been developed in an attempt to target microglial sensitization, in this case targeting the P2X4 (the ionotropic A TP receptor subunit-4) cascade. These show some efficacy, although only short-term outcomes have thus far been examined (∼7 days treatment), moreover these antibodies do not offer full relief from pain (Williams et al., 2019). Finally, many therapeutic antibodies only weakly permeate the blood–brain barrier, limiting analgesic targets to the periphery. Because chronic pain conditions are entrenched, repeated dosing may be required so antibodies that are able to pass the blood–brain barrier may be preferred. Together, antibodies appear to hold substantial potential as a method to neutralize pain mediators, a careful re-examination of targets may yet reveal exciting new therapies for pain.

A somewhat complementary approach is the recent development of targeting central spinal neurons using botulinum neurotoxin A conjugated to substance P or dermorphin. Using these approaches, specific classes of spinal neuron that express receptors for these ligands can be targeted, in this case NK1 or MOR expressing neurons respectively, and this can then inhibit inflammatory or neuropathic pain pathways. Whilst exceptionally promising these approaches have limited efficacy at present and do not seem able to restore pain to near baseline levels. However, the injection of dermorphin targeted botulinum toxin had an effect even when administered 2 weeks after nerve injury suggesting that silencing dorsal horn neurons is a therapeutic possibility for inhibiting pain that is already established (Maiarù et al., 2018). Together, these protein-based therapies have substantial potential for the future treatment of pain.

### Epigenetic Therapies

It has become increasingly apparent that certain epigenetic changes may underlie factors that maintain neuropathic pain (Descalzi et al., 2015), partly because they are likely to stabilize abnormal transcriptional profiles in the long term. Therefore, targeting these pain entrenching mechanisms may have therapeutic potential. It is thought that regulation of histone acetylation may play a role in the pathogenesis of pain. The pre-treatment of rats with histone de-acetylase (HDAC) specific inhibitors for instance is sufficient to attenuate neuropathic pain (Denk et al., 2013). However, the role of HDAC inhibitors is not limited to neuropathic injuries. Indeed, it is reported that inflammatory pain may be partially caused by a loss of GAD65 as a result of epigenetic repression. In this case, the deacetylation of histone residues around GAD65 leads to a decrease in expression through HDAC activity. Moreover, there is a requirement for GAD65 since the response doesn’t occur in GAD65 knockout mice (Zhang et al., 2011). Alternatively, the down regulation of potassium channels by G9a, a histone dimethyltransferase, after nerve injury appears to be involved in the chronic pain transition and this pathway is druggable with chaetocin (Laumet et al., 2015). A large issue with many of these pharmacological experiments is the lack of specificity of HDAC and methyltransferase inhibiting drugs, meaning it is unclear if the effects are mediated by HDAC modulation of epigenetic function. The agents commonly used to manipulate HDACs include valproate, sodium phenylbutyrate, and trichostatin A, which have other effects including GABA transporter inhibition, autophagic inhibition, tubulin stabilization and endoplasmic reticulum stress inhibition (Ricobaraza et al., 2009; Abematsu et al., 2010; Godena et al., 2014; Bondulich et al., 2016) that is mainly related to non-epigenetic roles of HDACs and drug promiscuity. Given that investigators rarely demonstrate the specificity of their effect, the mechanism by which these drugs improve pain symptoms remains unclear. Additionally, as of yet we do not understand enough about the specificity of epigenetic regulation within nociceptors and blunt instruments such as HDAC inhibition or histone methyltransferase inhibition are likely to have many of the problems associated with current pain therapies, namely specificity. As a result, until we are able to directly target the epigenetic state of nociceptors, therapies targeting epigenetic pathways remain unlikely until this can be overcome.

A potentially more fruitful approach is the direct modulation of the epigenetic state at specific loci. AKAP150 is reportedly involved in discogenic back pain, and epigenetic silencing using a dead cas9 tethered to a KRAB (kruppel associated box protein), that causes transcriptional silencing through depositing repressive H3K9me3 (Histone 3 Lysine 9 Trimethylated) marks can be achieved. This showed some promise in an *in vitro* model, but it has not been demonstrated *in vivo* (Stover et al., 2017).

### Nociceptor Specific Small Molecule Therapies

Since the discovery of congenital insensitivity to pain linked to Nav1.7 (*SCN9A*) mutations, Nav1.7 has emerged as a key drug target. Since Nav1.7 initially appeared to only be required for smell and pain (Cox et al., 2006), it was a tempting drug target with few potential side effects. Later evidence has shown that Nav1.7 may also contribute to the regulation of feeding and obesity through a centrally mediated mechanism, but given that its role in pain is thought to be peripheral, this may not have a significant impact on Nav1.7 inhibitors that do not pass the blood–brain barrier (Branco et al., 2016). Nav1.7 blockers have shown efficacy in clinical trials targeting erythromyalgia and diabetic neuropathy (Cao et al., 2016). However, they also highlight a considerable issue for the sensory neuron silencing approaches; where silencing of sensory neurons invariably causes neuropathy. Recent evidence suggests that neuropathy is a common consequence of Nav1.7 mutations that cause pain insensitivity and it remains to be seen if targeting nociceptors with long-term silencing agents also causes neuropathy in a more general sense (McDermott et al., 2019). If neuropathy is a general side-effect of targeting nociceptors, it will be important to balance pain relief with the risk of painless neuropathy. In a similar manner to Nav1.7 antagonists, membrane impermeant sodium channel blocker QX-314 (a lidocaine derivative) can show specificity to nociceptors when targeted using capsaicin agonism which dilates the TRPV1 pore enough to allow entry through the channel (Binshtok et al., 2007). If the problems of silencing can be addressed, these nociceptor-specific therapies offer substantial promise for pain. Similarly, because toxins can create pores for example staphylococcus aureus, these can also permit the specific entry of QX-314 (Blake et al., 2018) again targeting the analgesia to affected nerve fibers only. By targeting only nociceptors using small molecular therapies, there is considerable potential to create new, safer drugs. Moreover small molecules may have an easier route to market than genetic or cell therapies.

### Non-host Factors From Bacteria and Venomous Organisms

Toxins can cause substantial pain and suffering, in the case of toxin-induced pain, directly inhibiting toxin actions using molecular antidotes should be regarded as a potential therapy for pain. For example, the severe pain induced by venom from the box jellyfish, *Chironex fleckeri*, can be inhibited by targeting mechanisms linked to its ability to cause cell death specifically by treating with a cyclodextrin that removes cholesterol from cellular membranes is able to attenuate acute pain, sensitization and necrosis in mice (Lau et al., 2019). Whilst *Chironex fleckeri* envenomation remains rare, targeting snake envenomation using similar approaches may have potential for neglected tropical diseases that are having a growing impact owing to increased costs of anti-venoms and lack of antivenom efficacy in many cases (Isbister, 2010; Mohapatra et al., 2011; Brown, 2012). Similarly, in the case of bacterial infections, a frequent cause of pain is formylpeptide induced pain through the FPR1 receptor (Chiu et al., 2013), these can be targeted using antagonists to FPR1 such as boc-MLF (Chiu et al., 2013). Given that bacteria and venomous animals produce a host of toxins with diverse mechanisms of action, further investigation of the factors that cause pain and molecular therapies targeting these mechanisms have significant therapeutic potential. Moreover, similar processes may underlie the pain-causing potential of endogenous components of the inflammatory soup and targeting specific components using some of these strategies is feasible. Finally, pain caused by bacterial infection can inhibit immune function, targeting of these nociceptors is potentially critical to allowing the immune system to clear pathogens more effectively (Baral et al., 2018; Pinho-Ribeiro et al., 2018) and this highlights the importance of targeting pain not only for patient comfort but as a major pathological element of disease.

In addition to targeting these non-host factors, toxins and venoms seem to be an effective source of potential new analgesic compounds. Through an understanding of the components of venoms and toxins, many therapeutic compounds can be identified. For instance mycolactone from *Mycobacterium ulcerans* can elicit analgesia (En et al., 2008) through angiotensin receptor dependent potassium channel mediated hyperpolarization. This does not elicit nerve damage and therefore shows potential as an analgesic (Marion et al., 2014). Similarly, the identification of analgesic conotoxins derived from cone snail led to the development of conotoxins for pain. For instance, the intrathecal injection of Ziconotide can be used to inhibit severe chronic pain (Schmidtko et al., 2010). The delivery method also requires implantation of a intrathecal catheter which has practical limitations (Schmidtko et al., 2010). Currently, CNS side effects resulting from lack of specificity to pain neurons narrow the therapeutic window. Given that there are over 70,000 conopeptides these approaches have substantial promise both to find specific conotoxins for nociceptive neurons and to understand basic mechanisms of pain (Klimis et al., 2011; Adams et al., 2012; Mohammadi and Christie, 2015).

### Targeting Later Phases of Pain Sensitization

Whilst a lot of progress has been made developing therapies applied early in injury, prior to injury and during injury, more limited progress has been made in finding treatments for established neuropathic pain. Given that many patients have lived with pain for years, and patients who will go on to develop chronic pain are not necessarily easy to identify, it is unclear if the models we are currently using can identify effective treatments and moreover it is unclear the extent to which these pathologies are being effectively modeled in mice. With only a select few examples, in most studies the time frame of analgesia development is still limited to 3 weeks and most therapies are not tested for long-term efficacy. Examining therapies in the context of long-term treatments and efficacy is critical to developing new therapies that work in the clinic.

## How Can We Design Modern Treatments for Pain?

Chronic pain was historically seen as a symptom of a disease rather than a disease in its own right. Modern therapies for pain will need to consider pain as a disease in its own right and therefore we should take a mechanistic approach to its treatment. Pain should also be regarded as a highly heterogeneous disorder. In all probability, many pain mechanisms function together to create a complex pathological disorder. Studies must now look to address which of these mechanisms function together, and examine cross-talk between different pathways in greater depth. By understanding potential cross-talk and convergence between these mechanisms, we are likely to gain a better understanding of pain targets. Furthermore if we accept that pain is a heterogeneous condition, the stratification of patients by mechanism of action may be essential for effective treatments. Beyond pain itself we must not ignore the negative psychological and social effects of pain. We know that treating a disease alone is not necessarily enough to solve pain, we should accept that resolution of pain whilst ignoring the affective components of the disorder and the central components of the disease may be similarly ineffective.

Pain therapy itself is in crisis; addiction and poorly targeted drugs plague our current strategy for alleviation of chronic pain. Therefore, disease-modifying therapies which address central and peripheral sensitization are urgently needed. Whilst developing these new therapies we must be more careful to only recommend treatments with good evidence of efficacy obtained from randomized clinical trials and not subject patients to un-necessary and dangerous treatments. Finally, modern pain therapies should use targeting strategies that specifically target nociception and pain and move away from promiscuous drugs that have off-target central modulatory effects that underlie many of the side effects of analgesia. As we gain a deeper insight into the central mechanisms of pain disorders, we should be better able to treat pain diseases.

## Author Contributions

JM wrote the manuscript with assistance from TC, MW, and JNM contributed to editing and figure preparation. GN edited the manuscript and supervised the work.

## Conflict of Interest

JM and GN declare patent interests in pain therapies. The remaining authors declare that the research was conducted in the absence of any commercial or financial relationships that could be construed as a potential conflict of interest.
